# Mindwandering propensity modulates episodic memory consolidation

**DOI:** 10.1007/s40520-019-01251-1

**Published:** 2019-06-20

**Authors:** Samarth Varma, Atsuko Takashima, Li Fu, Roy P. C. Kessels

**Affiliations:** 1 Donders Institute for Brain, Cognition and Behaviour, Radboud University, Nijmegen, The Netherlands; 2 Max Planck Institute for Psycholinguistics, Nijmegen, The Netherlands; 3 Department of Medical Psychology, Radboud University Medical Center, Nijmegen, The Netherlands

**Keywords:** Memory consolidation, Retroactive interference, Mindwandering, Resting state, Working memory, Cognitive ageing

## Abstract

Research into strategies that can combat episodic memory decline in healthy older adults has gained widespread attention over the years. Evidence suggests that a short period of rest immediately after learning can enhance memory consolidation, as compared to engaging in cognitive tasks. However, a recent study in younger adults has shown that post-encoding engagement in a working memory task leads to the same degree of memory consolidation as from post-encoding rest. Here, we tested whether this finding can be extended to older adults. Using a delayed recognition test, we compared the memory consolidation of word–picture pairs learned prior to 9 min of rest or a 2-Back working memory task, and examined its relationship with executive functioning and mindwandering propensity. Our results show that (1) similar to younger adults, memory for the word–picture associations did not differ when encoding was followed by post-encoding rest or 2-Back task and (2) older adults with higher mindwandering propensity retained more word–picture associations encoded prior to rest relative to those encoded prior to the 2-Back task, whereas participants with lower mindwandering propensity had better memory performance for the pairs encoded prior to the 2-Back task. Overall, our results indicate that the degree of episodic memory consolidation during both active and passive post-encoding periods depends on individual mindwandering tendency.

**Electronic supplementary material:**

The online version of this article (10.1007/s40520-019-01251-1) contains supplementary material, which is available to authorized users.

## Introduction

Decline in episodic memory is a hallmark of cognitive ageing [1, 2]. An increasing number of studies have been focusing on factors responsible for such deficits as well as strategies to counteract it [3–5]. Particularly, in the area of episodic memory consolidation, a growing amount of research suggests that memory is better retained when participants rest quietly after learning. Instead, when learning is followed by cognitive tasks such as mental arithmetic [6], picture-naming [7] or autobiographical thinking [8], participants tend to forget recently learnt items. These findings have also been observed in cognitively unimpaired older adults [7], and in patients with (amnestic) mild cognitive impairment and Alzheimer’s dementia [9]. Rest is considered to be beneficial for memory consolidation [10, 11], partly due to the absence of interference from novel memory encoding/retrieval processes that are triggered by external stimulation.

However, in our previous studies with younger adults, we demonstrated that engaging in a 2-Back task in the post-encoding period leads to the same degree of memory consolidation as from a quiet wakeful rest state [12, 13]. This finding challenges the notion that rest or an absence of external stimulation is essential for successful consolidation. Unlike tasks that potentially trigger incidental memory encoding or retrieval [6–8], the 2-Back task could inhibit such memory processing due to its continuous working-memory (WM) demands and reduced hippocampal involvement [14, 15]. Therefore, it is likely that the 2-Back task might support consolidation by acting as a cognitive barrier against interference arising from memory processing cued by the environment.

Based on our findings in younger adults [12, 13], this study reexamines this question in healthy older adults. Changes in the hippocampal and prefrontal systems are closely associated with the decline of episodic memory in older adults [16, 17] and WM processing [18]. Particularly, performance on the *n*-Back task was found to be lower in older adults [19] and seems to decrease monotonically with advancing age [20]. Worse performance may be a result of less focus towards the task, causing higher chances of incidental autobiographical thinking, leading to interference with ongoing memory consolidation process [8]. However, on the other hand, older adults show a general over-recruitment of executive resources to compensate for age-related decline in visuospatial and sensory processing tasks [21, 22]. As such, task engagement may inhibit autobiographical thinking, and may support ongoing memory consolidation in a similar way to younger adults.

Ageing has also been associated with a reduction in mindwandering frequency [23]. It remains unclear whether this is a consequence of general cognitive decline [23], or that older adults simply have fewer task-unrelated thoughts [24]. However, there is strong evidence suggesting that mindwandering about events of the day may assist in episodic memory formation and consolidation, in a way similar to dreams [25]. For example, the replay of recently acquired memories may be facilitated by mindwandering about these experiences during offline periods like quiet wakeful rest, or when attention is tuned-out from external stimuli [23, 25]. On the other hand, laboratory experiments have also shown that mindwandering about memories unrelated to the learning experience, such as autobiographical thinking, can lead to forgetting of the recently learned items [8, 13]. The extent to which mindwandering supports consolidation might depend not only on the content of mindwandering, but on the difficulty of the concurrent task as well. Research has shown that mindwandering during simple tasks is considerably higher than during demanding tasks, as more cognitive resources are available for thoughts unrelated to the task when the task itself is easy to do [13, 23]. Accordingly, in terms of the current study, the difference between memory consolidation associated with the rest and 2-Back task conditions could be greatly affected by the mindwandering experienced by the participants.

Considering that such a decline in cognitive functioning and mindwandering may affect memory performance in older adults, the aim of this study is twofold. (1) Like in younger adults, does the 2-Back task support memory consolidation in older adults? (2) Would memory consolidation differ between rest and 2-Back conditions depending on individual mindwandering tendency? Similar to our previous study [12], older adults underwent two blocks of incidental encoding of word–picture pairs, each followed by 9 min of rest or a 2-Back task, ending with a recognition test. Executive functioning and mindwandering propensity were also measured separately. Following previous studies, and the compensatory account of cognitive ageing, we predicted that the 2-Back task might not cause interference to memory consolidation when controlling for the effect of cognitive decline. We further hypothesized that mindwandering propensity should modulate consolidation, as participants will have higher chances to mindwander during the rest delay period than the 2-Back delay period.

## Methods

### Participants

Thirty-eight older adults aged 62–78 years (16 women, *M*_age_ = 69.4, SD = 4.1) were recruited from Radboud University and randomly assigned to one of the two groups (RestEnd, 2BackEnd). All participants were native Dutch speakers, had no history of neurological or psychiatric illnesses (self-report) and scored higher than 25 on MMSE (*M *= 29.4, SD = 0.85, range = 26–30). Ten participants were excluded due to missing data, incorrect button presses and to balance the age and education between the two groups (blinded for outcome on the dependent variables). Two participants with extreme scores (± 2SD away from the mean) on questionnaire data were also removed from the analyses. This resulted in 13 participants in each group. Data from these remaining 26 participants (14 women, *M*_age_ = 68.7, SD = 4.2) were analyzed in this study (see Table 1). Nineteen participants had a pre-university/vocational level of education, while others attended some form of secondary school [26]. At the end of the experiment, participants received a monetary compensation.

**Table 1:** Memory scores represent the hits-false alarm rates related to each condition; D-prime scores represent normalized hits-false alarm rates related to each condition

Measurements	RestEnd group	2BackEnd group
Memory score: Rest condition	0.38 ± 0.22	0.40 ± 0.18
D-prime: Rest condition	1.21 ± 0.76	1.24 ± 0.60
Memory score: 2-Back condition	0.26 ± 0.19	0.50 ± 0.23*
D-prime: 2-Back condition	1.20 ± 0.78	1.97 ± 1.10*
Working memory score: 2-Back task	0.22 ± 0.24	0.47 ± 0.20*
Avg. RT: 2-Back task (seconds)	1.24 ± 0.45	1.22 ± 0.42
RNG score (range 0–1)	0.35 ± 0.04	0.32 ± 0.05
MWP score (max. 201)	109.69 ± 15.29	104.62 ± 17.22
Education level (1–5)	3.54 ± 0.66	3.92 ± 0.49
Age (years)	67.93 ± 3.89	69.54 ± 4.55

Working memory score represents the proportion of trials correctly identified as 2-Back trials-proportion of trials incorrectly identified as 2-Back trials. High scores in RNG or MWP measurements represent lower executive functioning capacity or higher mindwandering tendency, respectively

*Avg. RT*: *2*-*Back Task* (*s*) average reaction time in seconds during the 2-Back task, *RNGScore* score on the random number generation task, *MWPScore* score on the mindwandering propensity questionnaire, *Educationlevel* participants in each level of Dutch education (1: lower elementary school to 5: higher vocational training), *Age* participants age in years

*Significant differences (*p* < 0.001) across the order groups

## Material

### Stimuli

The experiment involved two encoding tasks and a combined associative recognition task. Similar to our previous study in younger adults [12], each encoding list consisted of unique 90 word–picture pairs. Words (adjectives) were generated using the MRC psycholinguistic database and translated into Dutch. Pictures (common objects, scenes or animals) were downloaded from various image databases on the Internet. To reduce influence of the saliency of the pictures to memory, the word–picture pairing was completely random for each condition and participant. For the recognition test, one-half of the trials consisted of half of the same pairs as seen during the encoding session (identical trials). The remaining word–picture pairs were recombined (recombined trials) leading to a total of 45 old pairs and 45 new pairs from each encoding list. No repetitions or new stimuli were presented during the recognition test.

### Post-experimental measures

To test the mindwandering propensity (MWP) of each participant, a short version of the imaginal process inventory questionnaire was created by selecting (and translating into Dutch) 40 questions that pertained to different components of daydreaming [27] (see Supplementary Material). The response possibilities were scored on a five-point scale. A high score on this questionnaire implied a higher tendency for mindwandering. Executive functioning ability was measured using a computerized random number generation (RNG) task [28], in which they randomly clicked on digits 1–9, laid out in a 3 × 3 grid on a screen. Performance on this task invokes WM processes such as maintenance of a set, monitoring previous responses, switching strategies as well as suppressing prepotent responses [28]. RNG task has been used as a measure of cognitive decline, as it positively correlates with a lack of strategy shifts and inhibition (for review, see [29]).

## Procedure

**Fig. 1:**
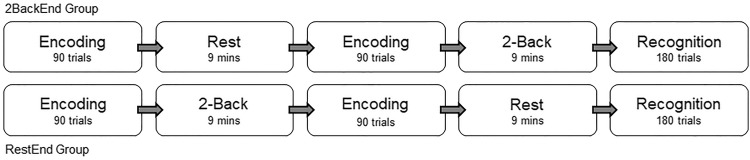
Incidental encoding task involved associative decision-making on word–picture pairs, followed by a 9 min consolidation period occupied by either rest or a 2-Back task. Subsequently, a surprise recognition memory test was administered by presenting 180 object–words pairs that were either identical to the encoding sessions or recombined. Participants were randomly allocated to either the 2BackEnd group or the RestEnd group in a counterbalanced manner

Similar to our previous study in younger adults [exp. 1; 12], the experiment involved two incidental encoding-delay blocks followed by a recognition test, post-experimental RNG task and MWP questionnaire. One of the delay periods consisted of quiet rest, whereas the other consisted of a 2-Back task; both lasting for 9 min each (see Fig. 1). Participants were randomly allocated to one of the two order groups (2BackEnd, RestEnd) in a counterbalanced fashion. One group (2BackEnd) received Rest in the first delay period and the 2-Back task in the second delay period, whereas the other group (RestEnd) had the order reversed. During each encoding trial, participants were asked to create an imaginative association between the word (e.g., “colourful”) and the picture (e.g., “helicopter”) displayed on the screen for a fixed duration of 4 s, and then, rate the vividness of their judgment on a scale of 1–3 using the keyboard within the next 5 s. After a button press or when the 5 s limit lapsed, the next trial began with a fixation-cross displayed for 0.5 s.

One of the 9-min delay periods (2-Back) comprised of a dynamic difficulty-adjusted 2-Back task [12]. For each trial, a random grayscale number (1 to 5) appeared in the middle of a screen for a maximum of 3 s, prompting the participants to either press “right” if they had seen the number two trials earlier, or press “left” otherwise. Following a keypress or after 3 s, the greyscale number turned green (correct) or red (incorrect or no response) for 300 ms showing a short feedback. Participants were acquainted with the task via a short practice at the beginning of the experiment. Depending on the cumulative performance of the participant at each trial, the duration of the next trial varied between 0.8 and 4.0 s. In this way, any change in participants’ skill was matched by a proportional change in task difficulty, thereby maintaining a sense of “flow” [30]. During the other delay period (Rest), participants rested in a quiet dark room for 9 min.

After the second encoding-delay period, the experimenter informed the participant about the surprise memory test. During the recognition test, all 180 trials were presented similar to the encoding trials except that the vividness judgment was replaced by a recognition question (“identical or recombined pair?”) lasting for a maximum of 5 s. On recognizing that a pair that was previously presented during one of the encoding sessions (i.e., “identical”), participants were asked for a confidence rating (“sure or unsure?”) in the next 3 s. Pairs from both encoding sessions appeared in random order during the recognition test. An optional break was allowed in the middle of the recognition test session.

Participants had received a short practice of all tasks before the experiment began. All experimental stimuli were presented using the PsychoPy presentation software [31].

## Analysis

Recognition trials for word–picture pairs to which a participant did not respond in the encoding phase were removed from the analyses. Since participants differed in their confidence ratings, we decided to include both sure and unsure trials in our analyses. Corrected memory performance scores were calculated as the difference between hit rate of identical trials and false alarm rate of recombined trials [32]. The resulting memory scores for each condition (associated with encoding prior to Rest or a 2-Back delay period) were added to a repeated-measures (RM) ANOVA as within-subject variables.

Based on our hypothesis related to the effect of mindwandering on post-encoding consolidation in the older age group, we added the mindwandering propensity score (MWP) to the RM-ANOVA as a covariate of interest. RNG task score was added as a covariate of no interest to regress out the effect of executive decline on memory performance. “Order” (levels: “RestEnd” and “2BackEnd”) was added as a between-group factor, since the degree of consolidation during each delay period could be affected by the order of the two encoding-delay blocks, and the time elapsed between encoding and recognition. All results were analyzed using IBM SPSS 23, at an alpha level of 0.05.

## Results

Participants in the “2BackEnd” group outperformed the “RestEnd” group on the 2-Back task performance; *t*(24) = 2.95, *p *= 0.007, despite matching on education levels (Mann–Whitney’s *U *= 60, *p *= 0.12), RNGScore (*t*(24) = − 1.68, *p *= 0.11), MWPScore (*t*(24) = − 0.79, *p *= 0.43), and age (*t*(24) = 0.97, *p *= 0.34) (Table 1). Accordingly, we expected that differences in the order of delay conditions across the order groups might influence the memory scores.

Descriptive tests between the experimental and demographic measures revealed a trend in correlation across participants between age and MWP score (Pearson’s *r *=* − *0.38, *p* = 0.058), suggesting a near-significant decline in mindwandering tendency with advancing age [23]. We also found a correlation between memory score associated with the 2-Back condition and 2-Back task performance (*r* = 0.54, *p* = 0.004). This suggests that a higher capacity to focus on the 2-Back task may be useful in inhibiting task-unrelated thoughts, thereby reducing interference to the ongoing consolidation of word–picture pairs encoded just prior to the 2-Back task [33]. Within each group (*N *= 13), however, these tests yielded different results. For the “RestEnd” group, the RNG score correlated positively with age and negatively with memory performance in both conditions, indicating that participants’ memory retention ability was affected by age-related cognitive decline. For the “2BackEnd” group, none of the aforementioned correlations were found. These results indicate that despite randomly allocating participants and equating the average age and education levels, the two order groups were not balanced. However, adding RNG score and order as nuisance variables in the RM-ANOVA may allow us to correctly interpret memory performance differences between the two delay conditions and the effect of mindwandering propensity.

One within (delay conditions) and one between (order) RM-ANOVA was tested on the recognition performance score. Consistent with our previous work in younger adults [12, 13], memory scores did not differ significantly between the two delay conditions; *F*(1, 22) = 0.33, *p *= 0.57. No main effect of order on average memory performance was found; *F*(1, 22) = 1.76, *p *= 0.19. However, we found a significant delay condition by order interaction effect; *F*(1, 22) = 12.31, *p *= 0.002. Posthoc paired *t* tests revealed that participants in the “RestEnd” group had higher memory performance associated with the Rest condition (*M* = 0.38, SD = 0.22) than the 2-Back condition (*M* = 0.26, SD = 0.19); *t*(12) = 2.99, *p* = 0.01, whereas in the “2BackEnd” group, memory performance associated with the Rest condition (Rest: *M* = 0.40, SD = 0.18) was lower than the 2-Back condition (*M* = 0.51, SD = 0.23); *t*(12) = − 2.28, *p* = 0.04. Posthoc independent-samples *t* tests showed that Rest condition did not differ across the two groups (RestEnd: *M* = 0.38, SD = 0.22; 2BackEnd: *M* = 0.40, SD = 0.18); *t*(24) = − 0.24, *p* = 0.81. However, in the case of the 2-Back condition, memory performance was significantly higher in the “2BackEnd” group (*M* = 0.51, SD = 0.23) than in the “RestEnd” group (*M* = 0.26, SD = 0.19); *t*(24) = 2.95, *p* = 0.007. In summary, this interaction effect seems to be driven by an advantage to the condition occurring in the second block, in particular for the 2-Back condition in the “2BackEnd” group.

**Fig. 2:**
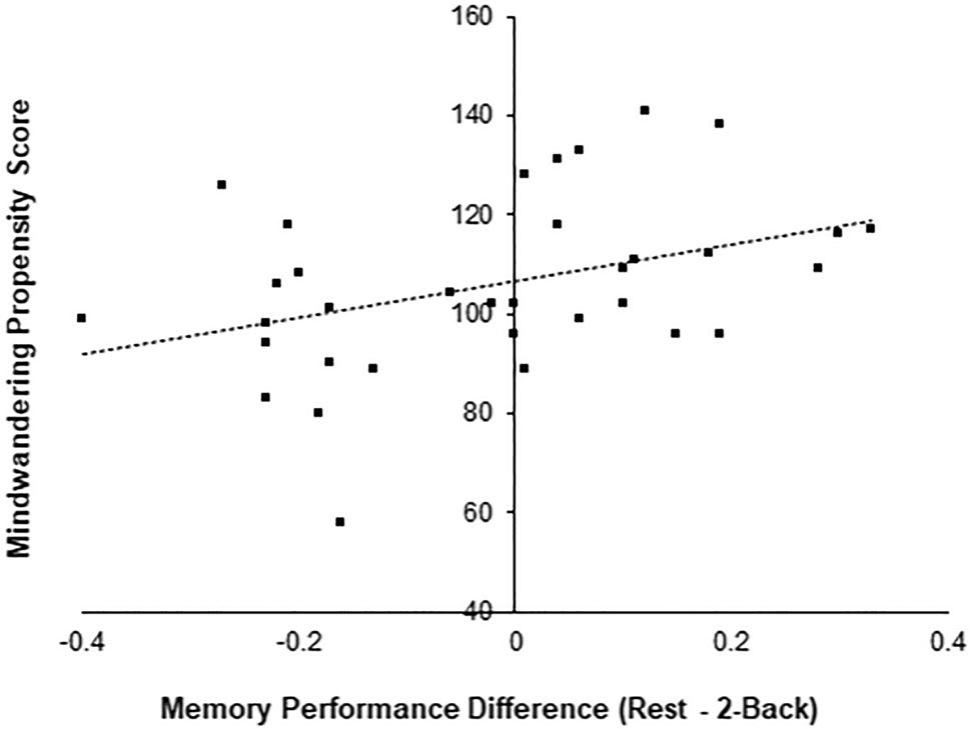
*Y*-axis corresponds to the mindwandering propensity score across all participants. The *X*-axis corresponds to the difference between associative memory recognition score of Rest condition minus 2-Back condition across all subjects. The plot represents the correlation between these measures (Pearson’s *r* = 0.41, *p* = 0.03), where each dot represents a single participant and the line represents best-fit linear trend

Further analysis did not show any significant interaction between the RNG score and memory performances of the two delay conditions: *F*(1, 22) = 0.45, *p* = 0.51. However, in line with our prediction, we found a trend towards a delay condition × MWP score interaction effect (*F*(1, 22) = 3.07, *p* = 0.09), indicating that the propensity to mindwander seems to affect the difference between the delay conditions irrespective of the order. To investigate this trend, we first ran a correlational test to check whether the MWP score modulates the difference in memory performance between the two delay conditions (Rest − 2-Back) regardless of the order. Our results showed a positive relationship between mindwandering propensity in the Rest condition over the 2-Back condition; Pearson’s *r* = 0.41, *p* = 0.03 (see Fig. 2). Running the correlation analysis separately for Rest and 2-Back conditions, however, did not show any significant effects (Rest: *p* = 0.33; 2-Back: *p* = 0.49). This finding suggests that relative to 2-Back condition, the retention of items learned prior to the Rest condition was better for participants who had a higher mindwandering propensity.

## Discussion

The principal aim of the current study was to investigate whether engaging in a post-encoding 2-Back task could promote memory consolidation in older adults to the same extent as a post-encoding rest period. Given that this finding was previously demonstrated in younger adults [12, 13], this study further investigated whether ageing-related reduction in executive functioning (RNG) and mindwandering (MWP) could affect memory consolidation. The results of our analyses showed that, (a) memory performance was no different when post-encoding-delay period was engaged in a 2-Back task or a quiet rest period similar to younger adults [12, 13] and (b) participants with higher mindwandering tendency performed better in the Rest condition as compared to the 2-Back condition, and vice versa. Along with the main results, we also found that (a) older age was associated with a decline in mindwandering tendency and (b) participants with a higher performance on the 2-Back task also showed better memory performance for items learned prior to the 2-Back task.

Previous studies have shown that older adults are susceptible to memory interference when the post-encoding period is filled with distracting tasks. However, similar to the case of younger adults [12, 13], the current study involving older adults shows that the overall retention of items learned prior to performing a cognitively demanding task (i.e., the 2-Back condition) does not differ significantly from the retention of items learned prior to a wakeful rest (Rest condition). This finding indicates that despite a consistently reported reduction in episodic and working-memory performance in older adults, engaging in a 2-Back task could support their memory consolidation similar to quiet wakeful rest. As indicated by the positive correlation between the 2-Back task performance and memory performance associated with the 2-Back condition, the effective recruitment of executive resources [34] during the 2-Back task might support memory consolidation by reducing interference arising from autobiographical thinking or environmental distractions [8].

Our results also show that the difference between the consolidation achieved during the Rest and 2-Back conditions was modulated by the mindwandering propensity of our participants. Participants with higher MWP scores benefitted more from the Rest condition than the 2-Back condition, whereas participants with lower MWP scores benefitted more from the 2-Back condition than the Rest condition. It seems to be the case that the directionality of the effect of mindwandering on memory consolidation depends on the content and the degree of mindwandering permissible in the post-encoding period [23]. In the case of post-encoding rest, it is possible that participant’s thoughts about the word–picture pairs may enhance memory retention [23]. However, if their thoughts are related to irrelevant autobiographical events for instance, memory consolidation of word–picture pairs may suffer from interference [8, 13]. As such, the degree of overall memory consolidation achieved during a rest period might be the result of the opposing effects of encoding-related and unrelated thoughts. Although our questionnaire did not measure the content of mindwandering during the delay period, we speculate that higher permissibility for mindwandering during the Rest condition allowed participants with higher mindwandering tendency to receive more support from thoughts related to the encoded material. Such participants were likely to have a high degree of autobiographical thoughts during the 2-Back task, causing poorer task and memory performance.

On the other hand, the reduction in permissibility of mindwandering in the 2-Back condition might benefit participants with lower mindwandering tendency. With lower mindwandering, these participants may have performed better at the 2-Back task [35], and achieve higher memory retention in the 2-Back condition, as indicated by the correlation between 2-Back task performance and memory score associated with the 2-Back condition. Lower mindwandering tendency also indicates that these participants were unlikely to benefit from learning-related thoughts during rest period. However, since we did not observe a direct correlation between mindwandering propensity and 2-Back task performance, our interpretation is only speculative.

Within the 2-Back group, the correlation between 2-Back task performance and memory performance might be the result of greater mindwandering suppression in high-performing participants, as compared to those who were easily distracted and performed poorly on the 2-Back task. The latter group of participants may have experienced mindwandering due to performance-related worries or introspection [28]. Being unrelated to the learning experience, these evaluative thoughts may have caused interference during the 2-Back task, in a way similar to the interference reported in the case of previously used interference tasks like psychometric tests, mental arithmetic, picture-search or autobiographical thinking tasks [6–8].

### Limitations

Although the degree of actual mindwandering prevalent during the consolidation conditions is highly relevant to our study, we did not use an experience-sampling measure to quantify such mindwandering to avoid uncontrolled interference effects. The post-experimental imaginal process inventory questionnaire (sIPI) provides a reliable indication of general mindwandering propensity in our participants, as it showed an expected decline of mindwandering with age, in line with previous literature [23], but it cannot be used to ascertain the content and degree of mindwandering during the delay periods. As such, future studies should include a short post-delay questionnaire to differentiate mindwandering that is related and unrelated to the learning experience, or probe thoughts within the delay periods. By obtaining such distribution of thoughts, the contribution of mindwandering to consolidation can be dissected more clearly [13].

Second, due to the relatively small number of participants in the study, and the perceivable difference across the groups in non-experimental measures, the results of this study must be interpreted with caution. However, in formulating our analyses, we have tried to neutralize any confounds related to order, cognitive decline or education by means of counterbalancing and the use of covariates in our statistical model. Since the main findings of the study reflect our prior results in younger adults [12], we speculate our interpretation to be meaningful.

## Conclusion

Similar to our previous work with young adults, this study involving cognitively unimpaired older adults showed that engaging in a post-encoding 2-Back task leads to the same degree of consolidation as post-encoding quiet wakeful rest. Furthermore, participants with higher mindwandering propensity showed better memory retention of items encoded prior to the rest period, whereas participants with lower mindwandering propensity retained more items learnt prior to the 2-Back task. Depending upon individual mindwandering tendencies, engaging in quiet wakeful rest or 2-Back task during the post-encoding period might serve as effective strategies to reduce ageing-related episodic memory decline.

## Electronic supplementary material

Below is the link to the electronic supplementary material.

Supplementary material 1 (XLSX 123 kb)
